# *mtlA,* a novel typing gene offering enhanced resolution for pandemic *Vibrio* species

**DOI:** 10.1128/spectrum.01105-24

**Published:** 2024-12-20

**Authors:** Lei Zhou, Danlei Liu, Zilong Zhang, Yongqiang Zhu, Huajun Zheng

**Affiliations:** 1 Shanghai-MOST Key Laboratory of Health and Disease Genomics, Shanghai Institute for Biomedical and Pharmaceutical Technologies (SIBPT), Fudan University, Shanghai, China; 2 Department of Microbiology and Immunology, School of Life Sciences, Fudan University, Shanghai, China; 3 Shanghai International Travel Healthcare Center, Shanghai Customs District PR China, Shanghai, China; Indian Institute of Technology Hyderabad, Hyderabad, Telangana, India

**Keywords:** *mtlA*, Vibrio, MLST, typing

## Abstract

The gene *mtlA*, encoding the mannitol transporter subunit IICBA of the phosphotransferase system, was the core gene with the greatest variability in *Vibrio parahaemolyticus* and could be used as a new typing marker in *Vibrio parahaemolyticus*. To expand its application, we performed an evolutionary analysis and found that the *mtlA* gene was present in nine phyla, 371 genera, and 1,662 species of bacteria. It is commonly found in pathogenic species of *Vibrio*, followed by *Paenibacillus*, *Mycolicibacterium*, etc. To investigate whether the *mtlA* gene could serve as a novel typing gene for whole *Vibrio* species, we compared the typing effect of the *mtlA* gene with multilocus sequence type (MLST) in 13 common pathogenic *Vibrio* species. The results revealed that the *mtlA* gene was present in all common pathogenic species, providing comparable or even higher typing resolution than MLST, especially in *Vibrio cholerae*, *Vibrio alginolyticus*, *Vibrio harveyi*, and *Vibrio anguillarum*, enabling accurate typing and traceability; however, many species lacked allelic profiles of MLST genes, making it impossible to obtain accurate STs. In summary, these findings validated the potential typing application of the *mtlA* gene in *Vibrio* species.

**IMPORTANCE:**

*Vibrio* is a collection of gram-negative bacteria characterized by possessing two chromosomes, which exhibit numerous shared biological and genomic traits. Around 13 species of *Vibrio* are known to cause infections in humans. Without appropriate medical interventions, the consequences of these infections can escalate to become life-threatening. Therefore, based on the prevalent characteristics of pathogenic *Vibrio*, it is imperative for us to classify and trace various *Vibrio* species that cause human illness and prescribe medication rationally to control the spread of diseases. In our investigation, we explored the prevalence and typing accuracy of the *mtlA* gene in *Vibrio*. Our findings revealed its ubiquitous presence across typical pathogenic *Vibrio* species, offering superior typing efficacy compared with MLST and more extensive implications.

## INTRODUCTION

To adapt to complex environments, bacteria have evolved sophisticated regulatory systems to maintain survival. For instance, when a specific metabolic substrate is lacking, the expression of genes encoding transporters and metabolic enzymes for that substrate is inhibited (1). Each organism exhibited carbon catabolite repression (CCR), with glucose typically at the top. Preferentially utilizable sugars such as glucose, fructose, or sucrose suppressed the synthesis of enzymes for secondary carbon sources when they were sufficient in the medium (2). The phosphoenolpyruvate (PEP):phosphotransferase system (PTS) in bacteria catalyzes the absorption and phosphorylation of various carbohydrates and plays a crucial role in CCR (3). PTS also participates in the metabolism of nitrogen, chemotaxis, potassium ion transport, and virulence regulation in certain pathogens (4). The activation of these regulatory functions depends on whether the PTS components are phosphorylated (4). Many bacteria and archaea can utilize carbon sources and their derivatives through PEP:PTS (5). The PTS consisted of one transmembrane protein and four soluble proteins. Cytoplasmic components include EI and HPr, which are involved in the uptake of all PTS substrates in most organisms, whereas EIIA, EIIB, and EIIC, as well as the transmembrane protein EIID in mannitol-type PTS, generally exhibit substrate specificity (4).

Mannitol is widely used as a low-calorie sweetener in the food industry and is distributed extensively in fungi, algae, and plants (6). MtlA, a mannitol transporter subunit IICBA of the PTS, can activate gene transcription related to biofilm formation and *vps* biofilm matrix exopolysaccharide synthesis in *Vibrio cholerae* through a mannitol transport-dependent pathway, providing it with a survival advantage in aquatic environments (7). In our previous study, we first identified the *mtlA* gene as a new typing gene of *Vibrio parahaemolyticus*, showing significantly greater resolution and typing efficacy than multilocus sequence typing (MLST) (8).

*Vibrio* species exhibit genomic diversity, but they all originate from marine environments and prefer to live in seawater. Their abundance reflected the temperature of the conditions (9). There are approximately 13 species of *Vibrio* that cause infections in humans and 16 species that affect marine organisms and aquaculture (10). For instance, *V. cholerae* causes cholera, a severe diarrheal disease transmitted through contaminated water or person-to-person contact. Without timely treatment, it can be life-threatening. Other *Vibrio* species, such as *V. parahaemolyticus*, *Vibrio alginolyticus*, and *Vibrio vulnificus,* also cause diseases in humans (11). In this study, we investigated the presence and typing resolution of the *mtlA* gene in *Vibrio*. We found that this gene was universally present in common pathogenic species of *Vibrio* and offered better typing efficiency for pathogenic *Vibrio* species than MLST.

## MATERIALS AND METHODS

### Retrieval and sequence download

The PTS mannitol transporter subunit IICBA protein was chosen as the research target. A search of the National Center for Biotechnology Information (NCBI) protein database was conducted using the term “PTS mannitol transporter subunit IICBA.” All reference proteins from each species were downloaded for analysis.

Thirteen pathogenic *Vibrio* species reported before 2024 were selected as the research objects. The sequences of *V. cholerae*, *V. parahaemolyticus*, *V. vulnificus*, *V. alginolyticus*, *Vibrio fluvialis*, *Vibrio mimicus*, *Vibrio metschnikovii*, *Vibrio metoecus*, *Vibrio harveyi*, *Vibrio anguillarum*, *Vibrio navarrensis*, *Vibrio furnissii,* and *Vibrio cincinnatiensis* were searched in the NCBI, and complete genomes were downloaded for typing analysis.

### Multiple sequence alignment

The MtlA protein sequences were imported into BioEdit 7.7.1, and then, the ClustalW multiple alignment method was used for alignment. The output was edited in Jalview 2.11.3.2 (12).

### Construction of a phylogenetic tree of MtlA in bacteria

The alignment results were imported into IQTREE2 (13). The maximum likelihood (ML) method was chosen for constructing a phylogenetic tree with the following parameter settings: bootstrap: 5,000.

### Typing

From all complete genomes, MLST genes were extracted and uploaded to the PubMLST database to identify STs. The *mtlA* gene was also extracted, and the BLASTCLUST (parameter: sequence identity 100%) command was used for genotyping.

### Evolutionary analysis of the *mtlA* gene in *Vibrio*

All the *mtlA* gene sequences of pathogenic *Vibrio* species were aligned using MAFFT v6.864b (14). The alignment was subjected to the neighbor-joining (NJ) method to construct a phylogenetic tree.

## RESULTS

### Distribution of MtlA in bacteria

**Fig 1:**
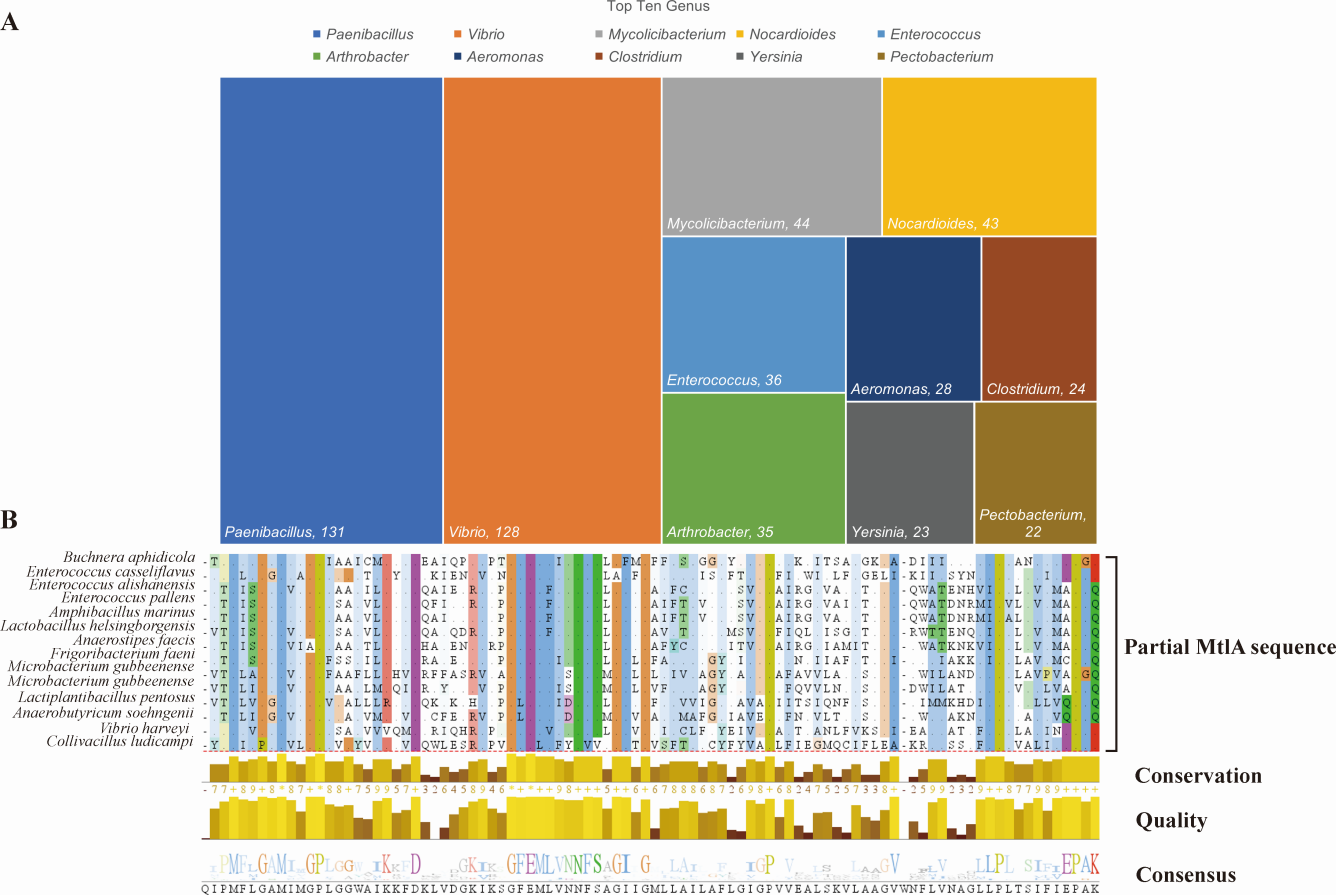
The top 10 *genera* and multiple alignment. (**A**) The top 10 genera associated with MtlA. The top genera included *Paenibacillus* (131), *Enterococcus* (36), and *Clostridium* (24) from Firmicutes; *Mycolicibacterium* (44), *Nocardioides* (43), and *Arthrobacter* (35) from Actinobacteria; and *Vibrio* (128), *Aeromonas* (28), *Yersinia* (23), and *Pectobacterium* (22) from Proteobacteria. (**B**) Multiple alignment of MtlA. Only part of the 1,662 species were shown, and the species names are located on the left, and the amino acids are numbered from left to right.

A search for MtlA protein in the NCBI database revealed a total of 62,276 items, including eight records in animals, seven in plants, nine in fungi, eight in archaea, five under viruses, two under protists, and 62,237 from bacteria. Upon verification using reference proteins from each species, it was found that apart from the bacteria, none of the MtlA proteins from the other taxonomies matched the PTS mannitol transporter subunit IICBA protein. Therefore, MtlA proteins were mainly distributed in nine phyla (*Actinomycetota*, *Bacillota*, *Pseudomonadota*, etc), 371 genera, and 1,662 species. The top 10 genera containing MtlA included *Paenibacillus* (131), *Enterococcus* (36), and *Clostridium* (24) from *Bacillota; Mycolicibacterium* (44), *Nocardioides* (43), and *Arthrobacter* (35) from *Actinomycetota*; and *Vibrio* (128), *Aeromonas* (28), *Yersinia* (23), and *Pectobacterium* (22) from *Pseudomonadota*, as shown in Fig. 1A.

A total of 165 *Vibrio* species were identified. Among them, 13 species causing human infections included *V. cholerae*, *V. vulnificus*, *V. alginolyticus*, *V. fluvialis*, *Vibrio harveyi*, etc. Finally, 377 complete genome sequences of the 13 species were downloaded for analysis (Table 1).

**TABLE 1:** The number of complete genomes of common pathogenic *Vibrio* species

Common pathogenic *Vibrio* species	No. of complete genomes
*V. cholerae*	132
*V. parahaemolyticus*	75
*V. vulnificus*	28
*V. alginolyticus*	44
*V. fluvialis*	19
*V. harveyi*	28
*V. anguillarum*	16
*V. metschnikovii*	3
*V. navarrensis*	2
*V. metoecus*	5
*V. mimicus*	7
*V. furnissii*	13
*V. cincinnatiensis*	4

### Multiple sequence alignment

The MtlA protein sequences representing 1,662 species were imported into BioEdit for multiple sequence alignment (partial alignment results are shown in Fig. 1B, and the entire alignment result is listed in Table S1). Conserved residues were identified at positions 190 (leucine, Leu, L), 191 and 192 (asparagine, Asn, N), 227 and 255 (glycine, Gly, G), 258 (glutamic acid, Glu, E), and 262 and 268 (proline, Pro, P), with 100% identity. This finding suggested that these positions may be relevant to the biological function of MtlA in the transmembrane transport of mannitol.

Furthermore, we identified 25 positions that are highly conserved, with 100% identical amino acids present in *Vibrio* and the majority of other species (Table S2). For example, at position 127, phenylalanine is prevalent across most genera, including *Vibrio*, whereas in *Olsenella profusa,* it is replaced by leucine. Likewise, at position 137, alanine is uniquely found in *Anaerococcus hydrogenalis*.

### Phylogenetic analysis of MtlA in bacteria

**Fig 2:**
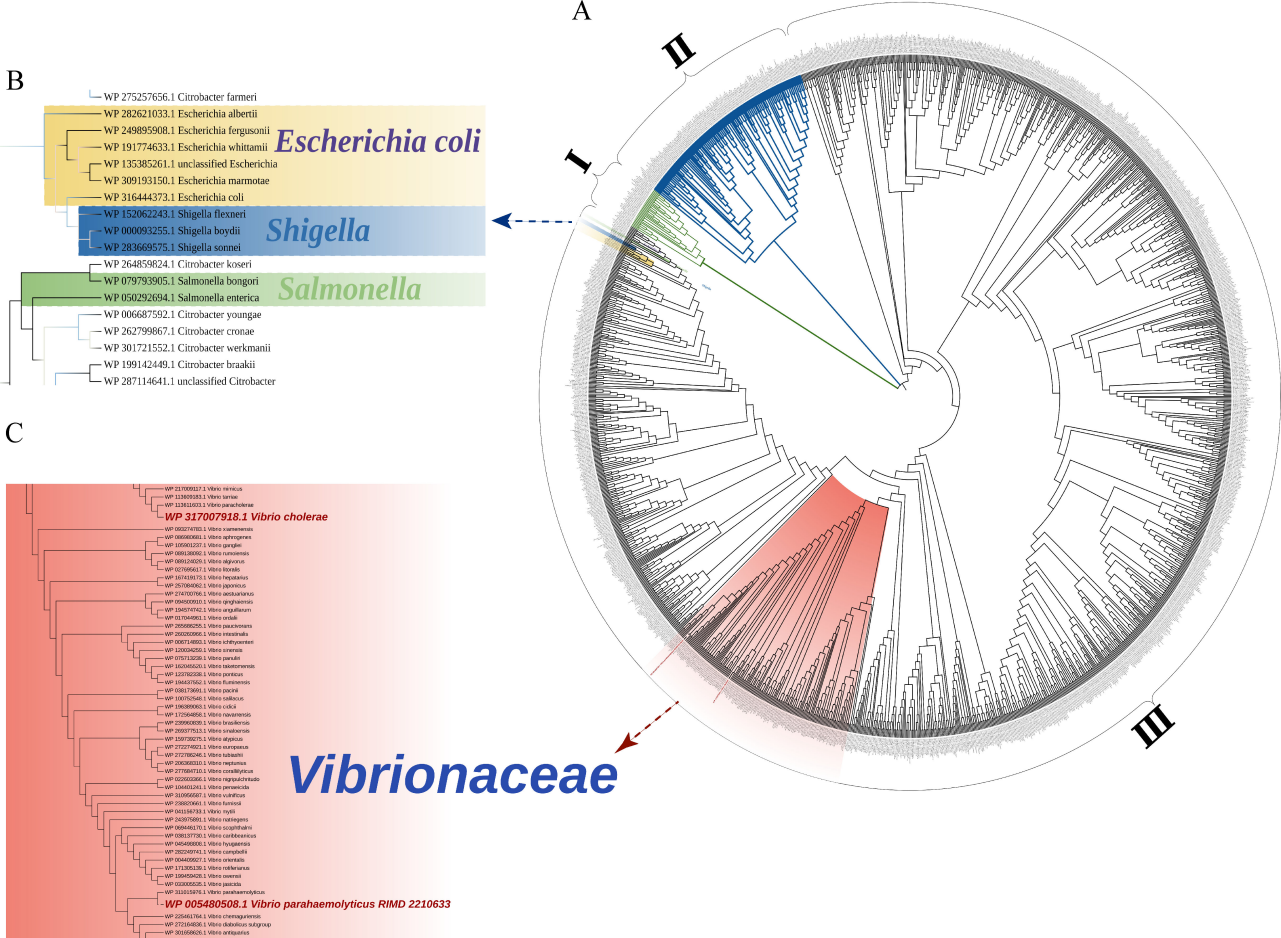
Maximum likelihood phylogenetic tree based on MtlA. (**A**) This is the complete phylogenetic tree of all sequences in bacteria; (**B** and **C**) these represent the local enlargement of the MtlA phylogenetic tree for common gastrointestinal pathogenic bacteria.

By constructing a phylogenetic tree of 1,662 protein sequences, we observed that all sequences clustered into three categories (Table S3) (Fig. 2A): class I included 34 species, such as *Cutibacterium* species from *Actinomycetota* and *Enterococcus* species from *Bacillota*; class II contained 152 species, including *Clostridium* species from *Bacillota*, *Selenomonas* species, and a small number of *Paenibacillus* species; and class III was the largest unit, mainly including *Vibrio* species from *Pseudomonadota*, *Paenibacillus,* and *Enterococcus* from *Bacillota*, *Mycolicibacterium,* and *Nocardioides* from *Actinomycetota*, totaling 1,446 species. From the phylogenetic tree, we discovered that other common gastrointestinal pathogens such as *Escherichia coli*, *Shigella*, and *Salmonella* had a relatively distant evolutionary relationship with the *Vibrio* genus, with low homology to *Vibrio* MtlA (Fig. 2B and C). Therefore, we inferred that the *mtlA* gene did not exhibit affection for other gut bacteria but was instead a specific typing gene for the genus *Vibrio*.

*V. parahaemolyticus*, belonging to *Vibrionaceae*, was clustered within class III, which also included other common intestinal *Vibrio* species, such as *V. cholerae*, *V. vulnificus*, *Vibrio anguillarum*, and *Vibrio mytili*. The MtlA sequences of 13 *Vibrio* species exhibited a certain degree of similarity (identical sites 59.5%). The MtlA sequences from other *Pseudomonadota*, including *Tolumonas lignilytica* of the *Pseudomonadaceae* family, *Mannheimia succiniciproducens* of the *Pasteurellaceae* family, and *Zobellella iuensis* of the *Alteromonadaceae* family, exhibited a close phylogenetic relationship with those found in *Vibrio*. Other genera, such as *Cutibacterium* and *Enterococcus* in *Actinomycetota* and *Anaerococcus* in *Bacillota*, were distantly related to *Vibrio*. This indicated that the phylogenetic distances of MtlA aligned with the evolutionary relationships of various species, without horizontal gene transfer (HGT) events.

Common gastrointestinal pathogens also included *Shigella*, *Salmonella*, and *E. coli*, and most of these strains harbored MtlA. Although these genera shared genetic proximity, their homology with *Vibrio* MtlA was low. Therefore, the typing effect of *mtlA* screened in *V. parahaemolyticus* in our previous study could be applicable to typing gastrointestinal pathogens, especially *Vibrio*.

### Typing of the gene *mtlA* and MLST

MLST and *mtlA* gene typing was conducted using complete genomes of human pathogenic *Vibrio*. The typing results of these two typing methods for *V. cholerae* are provided in Table S4. We discovered that MLST could be used to categorize 132 *V*. *cholerae* genomes into 31 STs, with 24 STs (77.41%) containing only one strain, such as ST5, ST8, ST14, ST70, ST746, and ST1257. The *mtlA* gene showed a greater resolution than MLST, and the 132 genomes were classified into 45 clusters, with 35 (77.78%) consisting of one genome. Furthermore, from the protein level, MtlA can divide 132 genomes into 37 clusters, with 25 of them containing one genome. Therefore, the resolution of the *mtlA* gene is superior to that of the traditional MLST in *V. cholerae* typing, whether it is considered from the nucleic acid or the protein level. Subsequently, we compared the two typing methods for *V. vulnificus*, as shown in Table S5. MLST of 10 housekeeping genes revealed that 28 *V*. *vulnificus* genomes could be divided into 18 STs, with eight genomes lacking definitive allelic profiles of some genes, such as strain 07–2444, which only had accurate allelic profiles for *gyrB*, *mdh*, and *metG* and was thus unable to provide STs. The single gene *mtlA* in nucleotide form could be used to classify all the genomes into 17 clusters, distinguishing 07–2444 from other strains and forming a unique type.

Other *Vibrio* species were typed using the same four housekeeping genes (*gyrB*, *pyrH*, *recA*, and *atpA*) for STs in the *Vibrio* spp. module of PubMLST. We revealed that compared with those of the other *Vibrio* species, the allelic profiles of the four housekeeping genes of *V. alginolyticus*, *V. harveyi*, and *V. anguillarum* were relatively complete. MLST divided the 44 genomes of *V. alginolyticus* into 16 STs, 13 of which were unique. In contrast, the single gene *mtlA* classified the 44 strains into 29 clusters, with 22 containing one strain. However, because different codons code for the same amino acid, the resolution of *mtlA* at the protein level was not as good as that of traditional MLST. Therefore, this suggested that high-resolution typing of the gene *mtlA* should be based on nucleic acid sequence. All 16 *V*. *anguillarum* genome sequences were typed as ST163 by MLST, whereas the *mtlA* gene distinguished them into eight clusters, with five single clusters. MLST genes were incomplete in 28 *V*. *harveyi* strains, with only three strains having definitive STs (ST241), whereas the *mtlA* gene was present in all complete genomes and classified into 23 clusters, with 20 strains forming unique clusters (Table S6).

Our research results indicated that the resolution of the *mtlA* gene not only fully covered the typing of the four housekeeping genes used in MLST but also distinguished strains that cannot be differentiated by MLST. For example, the housekeeping genes used in traditional MLST were fully present in only nine strains (56.25%) of *V. anguillarum* and can only be classified into one type, ST163. In contrast, the *mtlA* gene was present in 15 strains (93.75%) and provided a higher resolution that covered MLST, allowing differentiation of strains within ST163, such as 87-9-116 and 75, into different types (Table S6). These results indicated that the *mtlA* gene or MtlA protein not only widely existed in *Vibrio* species but also achieved higher resolution than MLST in most species, thus showing promising application prospects.

In the remaining species, such as *V. fluvialis*, *V. harveyi*, *V. metschnikovii*, *V. navarrensis*, *V. metoecus*, *V. mimicus*, and *V. cincinnatiensis*, only the *gyrB* or *aptA* genes had definitive allelic profiles in PubMLST, making it difficult to determine specific STs. For example, in *V. fluvialis*, strains IDH05335 and I7A had only the *gyrB* gene with a definitive allelic profile for MLST, whereas the remaining three gene profiles were unclear, rendering it impossible to ascertain the ultimate STs. However, the *mtlA* gene was identified in these strains and can completely differentiate them into two separate types. Therefore, the *mtlA* gene was present in all the genomes of these *Vibrio* species and had good resolution, making it suitable for typing and tracing purposes.

### Analysis of the *mtlA* gene in the *Vibrio* phylogeny

**Fig 3:**
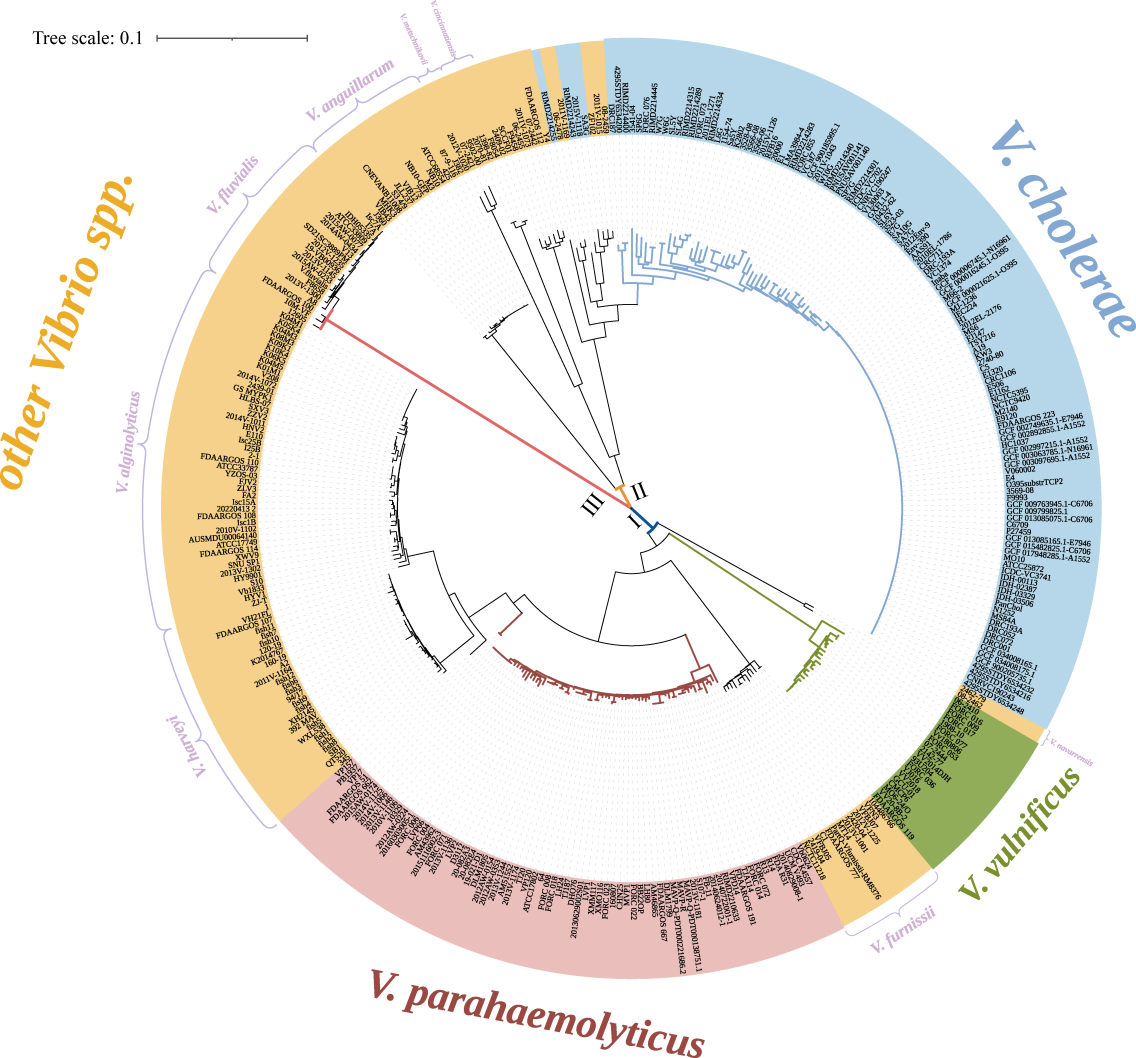
Phylogenetic tree of *Vibrio* strains constructed based on the *mtlA* nucleotide sequence. The phylogenetic tree is divided into three categories, each represented by solid lines of different colors. Category I is represented by blue solid lines, category II by orange, and category III by red. On the outside of the labels, the blue strip represents *V. Cholerae*, the pink strip represents *V. parahaemolyticus*, the green strip represents *V. vulnificus*, and the yellow strip represents the other strains.

A phylogenetic tree was constructed using the *mtlA* gene of commonly encountered *Vibrio* species (Fig. 3). The entire evolutionary tree primarily clustered into three groups. From the tree, *V. parahaemolyticus*, *V. vulnificus*, *V. alginolyticus*, *V. harveyi*, and *V. furnissii* formed one group; *V. cholerae*, *V. anguillarum*, *V. alginolyticus*, *V. cincinnatiensis*, *V. mimicus*, and *V. metoecus* were closely related; and *V. fluvialis* formed a separate group. We found that strains of the same species generally clustered together, suggesting that although the *mtlA* gene exhibited high variability, there was still a certain degree of conservation within species.

## DISCUSSION

Since the advent of microbiology by Antonie van Leeuwenhoek, who improved microscope and bacterial observation, bacterial classification has long relied on morphology (15). From the 1960s to the 1980s, chemical, numerical taxonomy, and DNA-DNA hybridization methods were developed (16–18). From 1980 onward, genetic analysis, MLST, average nucleotide identity, and whole-genome scale analysis have gradually emerged (19).

*Vibrio* encompasses a group of gram-negative rod-shaped bacteria and possesses two chromosomes that share many common biological and genomic features (9, 10). There are 165 species of *Vibrio*, approximately 13 of which commonly cause human infections, including *V. cholerae*, *V. parahaemolyticus*, *V. vulnificus*, and *V. alginolyticus*. The distribution of pathogenic *Vibrio* species is increasing in temperate waters of the Northern Hemisphere, including regions such as the Baltic Sea in Northern Europe (20). Currently, the Centers for Disease Control and Prevention (CDC) and the World Health Organization (WHO) have collected and monitored global epidemiological data on important pathogens, but only a few countries have dedicated surveillance for *Vibrio*. There is still a lack of a global systematic monitoring network and comprehensive databases for typing species within *Vibrio* (21). The PubMLST database only provided independent typing modules for four *Vibrio* species, *V. cholerae*, *V. parahaemolyticus*, *V. vulnificus*, and *V. tapetis*, whereas other pathogenic species, such as *V. anguillarum*, *V. alginolyticus*, *V. cincinnatiensis*, *V. mimicus*, and *V. metoecus,* were grouped under *Vibrio* spp. and utilized the same MLST genes.

In our former research, we initially discovered the *mtlA* gene as a novel typing marker for *V. parahaemolyticus*, demonstrating markedly superior resolution and typing efficiency compared with MLST (8). Upon checking the reference genomes of MtlA sequences in each species, we determined that this protein was present only in bacteria. In this study, MtlA was found in nine phyla, 371 genera, and 1,662 species of bacteria. The analysis revealed that the *mtlA* gene was mainly distributed in *Bacillota,* such as *Paenibacillus*, *Enterococcus*, and *Clostridium; Actinomycetota,* such as *Mycolicibacterium* and *Nocardioides*; and *Pseudomonadota,* such as *Vibrio* and *Aeromonas*, which are currently the most extensively studied genera. We conducted a conservative analysis of the MtlA sequence in 1,662 species and detected eight totally conserved sites (100% identity), including Leu at position 190, Asn at positions 191 and 192, Gly at positions 227 and 255, Glu at position 258, and Pro at positions 262 and 268 (Fig. 1B). This indicated that these sites may be correlated with the biological function of the transmembrane transport of mannitol.

In addition, another 25 amino acids were totally conserved in *Vibrio* and were mutated in only about 1% of the 1,662 species, further validating the structure conservation and functional importance of the MtlA protein. For example, at position 69, 98.86% of the species is glutamine, a polar amino acid, and 1.08% is alanine, a nonpolar, hydrophobic amino acid. The change between the two amino acids may lead to changes in local structure, especially in terms of surface charge and hydrogen bond-forming capability. Previous research has indicated that in some transmembrane proteins, changes in amino acids may induce more severe structural and functional abnormalities (22).

HGT is one of the main ways by which bacteria exchange genetic material to acquire exogenous genes from other strains, thereby resulting in new biological functions (23). Early studies reported that *Pseudomonas aeruginosa* can utilize the T6SS to transfer toxins to neighboring bacteria through contact (24). In this study, a phylogenetic tree was constructed using 1,662 MtlA protein sequences, revealing that strains belonging to the same phylum were closely related, whereas those from different phyla had distant evolutionary distances, indicating that the *mtlA* gene had not undergone HGT. Additionally, MtlA is commonly found in pathogenic species of the genus *Vibrio*.

In our study, we downloaded 13 human pathogenic *Vibrio* reference genomes from the NCBI and found that the *mtlA* gene was present in all common pathogenic species. The resolution of the *mtlA* gene was comparable with that of MLST, and the *mtlA* gene even exhibited higher resolution in *Vibrio* strains such as *V. cholerae*, *V. alginolyticus*, *V. harveyi*, and *V. anguillarum* (Tables S4 and S6). The high-resolution typing methods should place greater emphasis on nucleic acid sequences, as they can improve resolution by identifying subtle genetic variations, rather than relying on the encoded amino acid sequences. For example, the gene *mtlA* categorized the 44 *V*. *alginolyticus* genomes into 29 clusters, of which 22 clusters included only one strain each. However, the amino acid sequence of *mtlA* can only divide the 44 genomes into 13 clusters, with only 10 clusters containing just one strain each. From the evolutionary tree, we observed that strains belonging to the same species tended to group closely together. This indicated that despite the high variability of the *mtlA* gene, there remains a level of conservation within *Vibrio* species (Fig. 3). We also discovered that the *V. cholerae* and *V. metoecus* strains might have a close genetic distance, such as the close proximity between SA3G (*V. cholerae*) and ZF102 (*V. metoecus*). Earlier studies have shown that these two species were both isolated from an unaffected brackish coastal pond, where they interacted through HGT and were difficult to distinguish (25, 26). However, here, we can differentiate them using the single gene *mtlA*, achieving precise typing and tracing. Therefore, in addition to the ability of the *mtlA* gene to differentiate closely related strains of *V. parahaemolyticus* in a previous study, its high resolution has also been applied to other *Vibrio* species.

In summary, we found that the MtlA protein was distributed among nine phyla and 371 genera of bacteria and was especially widely distributed in common pathogenic *Vibrio* species. Moreover, during its long evolutionary process, there has been no significant HGT; thus, we speculated that the *mtlA* gene might serve as a novel typing and tracing gene specific to *Vibrio*. Then, we applied this efficient typing gene, *mtlA*, which has been validated in *V. parahaemolyticus*, to common pathogenic *Vibrio* species. We discovered that the *mtlA* gene was present in all the reference genomes of common *Vibrio* species and performed better than MLST, demonstrating higher resolution and the ability to achieve typing traceability.

### Conclusion

Based on our previous study, we further investigated the distribution of MtlA in bacteria and revealed its notable prevalence in *Vibrio*, *Paenibacillus, Mycolicibacterium*, etc. This suggested the potential applicability of the *mtlA* gene in typing various pathogens. Unlike MLST, whose typing genes are often lacking in certain *Vibrio* species, the *mtlA* gene is present in all common pathogenic *Vibrio* species. Its typing efficacy was comparable with that of MLST, and it even exhibited higher resolution in *V. cholerae*, *V. alginolyticus*, *V. harveyi*, and *V. anguillarum*.

## Data Availability

The data underlying this article are available at National Center for Biotechnology Information (NCBI) (https://www.ncbi.nlm.nih.gov/).

## SUPPLEMENTAL MATERIAL

Table S1 spectrum.01105-24-s0001.xlsx The entire alignment result of MtlA protein in bacteria.

Table S2 spectrum.01105-24-s0002.xlsx Highly conserved amino acid residues.

Table S3 spectrum.01105-24-s0003.xlsx 1,662 sequences of MtlA protein clustered into three categories.

Table S4 spectrum.01105-24-s0004.xlsx The typing result of MLST and gene mtlA in *V. cholerae*.

Table S5 spectrum.01105-24-s0005.xlsx The typing result of MLST and gene mtlA in *V. vulnificus*.

Table S6 spectrum.01105-24-s0006.xlsx The typing result of MLST and gene mtlA in *Vibrio* spp.

ASM does not own the copyrights to Supplemental Material that may be linked to, or accessed through, an article. The authors have granted ASM a non-exclusive, world-wide license to publish the Supplemental Material files. Please contact the corresponding author directly for reuse.
